# Total hip arthroplasty in patients with vertebral compression fracture is associated with poor clinical outcomes – retrospective analysis on 453 cases

**DOI:** 10.1186/s40634-023-00618-8

**Published:** 2023-05-24

**Authors:** Chin-Hsi Chen, Yaichiro Okuzu, Koji Goto, Yutaka Kuroda, Toshiyuki Kawai, Shuichi Matsuda

**Affiliations:** Department of Orthopaedic Surgery, Kyoto University Graduate School of Medicine, 54 Shogoin-Kawahara-Cho, Sakyo-Ku, Kyoto, 606-8507 Japan

**Keywords:** Vertebral compression fracture, Total hip arthroplasty, Low back pain, Clinical outcome

## Abstract

**Purpose:**

Total hip arthroplasty (THA) is increasingly performed in older adults, and the prevalence of vertebral compression fracture (VCF) increases with age. We aimed to investigate the clinical outcomes of THA in patients with VCF.

**Methods:**

We reviewed the records of 453 patients who underwent THA at our institution between 2015 and 2021. We classified patients into those with and without VCF. VCF was identified using preoperative upright whole-spine radiographs. Spinal parameters, preoperative and 1-year postoperative clinical outcomes of the Harris hip score (HHS), Oxford hip score (OHS), and visual analog scale (VAS) for low back pain (LBP) were assessed. Furthermore, propensity score-matched cohorts for age, sex, body mass index, and spinal parameters were created, and the clinical outcomes were compared between the two groups.

**Results:**

Among the 453 patients, 51 (11.3%) with VCF and 402 without VCF were identified. Before matching, patients with VCF were older (*p* < 0.01), had sagittal spinal imbalance (*p* < 0.01), and had worse clinical outcomes pre- and postoperatively. After matching 47 patients in both groups, patients with VCF had worse HHS (*p* < 0.05), especially regarding support and distance walked, and worse VAS scores for LBP (*p* < 0.05) pre- and postoperatively. However, the improvements in scores were not significantly different between the groups.

**Conclusions:**

HHS, especially regarding support and distance walked, and VAS scores for LBP were poorer in patients with VCF preoperatively and 1-year postoperatively. Our findings suggest that hip surgeons should evaluate not only spinal alignment but also the presence of VCF before performing THA.

**Level of evidence:**

Level III, Retrospective cohort study.

## Background

Total hip arthroplasty (THA) is an effective treatment for degenerative changes and pain in the hip joints. The volume of primary THAs performed has increased over the past several decades [15, 17, 25, 26] and will continue to increase until 2030 [16, 18, 26]. The number of THAs for older adults will rise by approximately twice as much in 2030 as in 2020 [18], but has a higher risk of complications, including dislocation, periprosthetic fracture, and infection [20, 28]. Hip surgeons should have greater knowledge of the characteristics of older adults who undergo THA.

Osteoporotic vertebral compression fractures (VCFs) are common in older adults. Globally, the prevalence of radiographic vertebral fractures has increased with age [24]. Many studies have shown that VCFs lead to back pain, poor physical performance, and lower health-related quality of life (HRQoL) [1, 2, 13]. Recent studies have reported the relationship between spinal disorders and clinical outcomes of THA. Sagittal spinal imbalance, spinal fixation surgery, lumbar spinal disorders, and low back pain (LBP) have adverse effects on the clinical outcomes of THA [9, 19, 21, 23]. However, little evidence exists regarding the clinical outcomes of THA in patients with VCF. Furthermore, the number of THAs in patients with VCF will increase in the future owing to the increased number of THAs in older adults. Therefore, we aimed to investigate the clinical outcomes of THA in patients with VCF.

## Patients and methods

### Ethics

This study was approved by the relevant Institutional Review Board and conducted in accordance with the World Medical Association Declaration of Helsinki. Written informed consent was obtained from the retrospective studies of all study participants.

### Study design

**Fig. 1:**
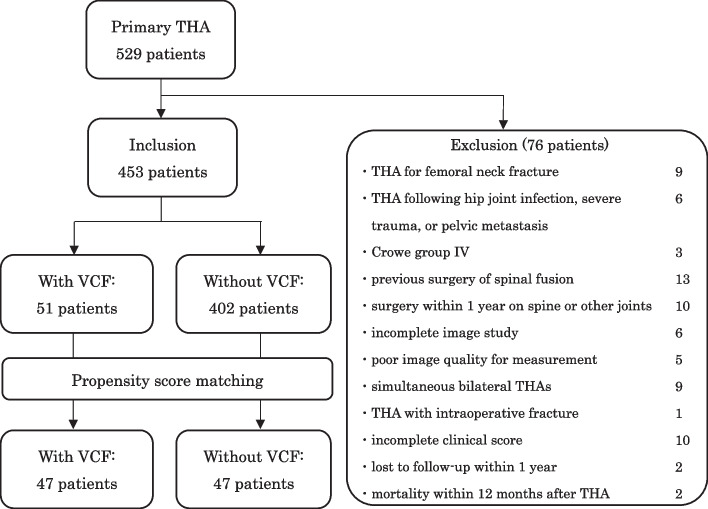
Flowchart of patient inclusion, classification, and propensity score matching. Propensity score matching was performed for age, sex, body mass index, C7 sagittal vertical axis, and pelvic incidence minus lumbar lordosis. THA, total hip arthroplasty; VCF, vertebral compression fracture

This was a retrospective cohort study. We reviewed 529 consecutive patients who underwent primary THAs at our institution between November 2015 and July 2021. All patients were registered in our database. This study finally included 453 patients following the exclusion of 76 patients for the following reasons (Fig. 1): THA for femoral neck fracture, Crowe IV dysplasia [7], hip joint infection, severe lower limb trauma, metastasis of the hip, previous spinal fusion surgery, surgery of other joints or spine within 1 year, incomplete image study, poor image quality for measurement, simultaneous bilateral THAs, THA with intraoperative fracture, incomplete clinical functional assessment, loss to follow-up, or death within 12 months after THA.

**Fig. 2:**
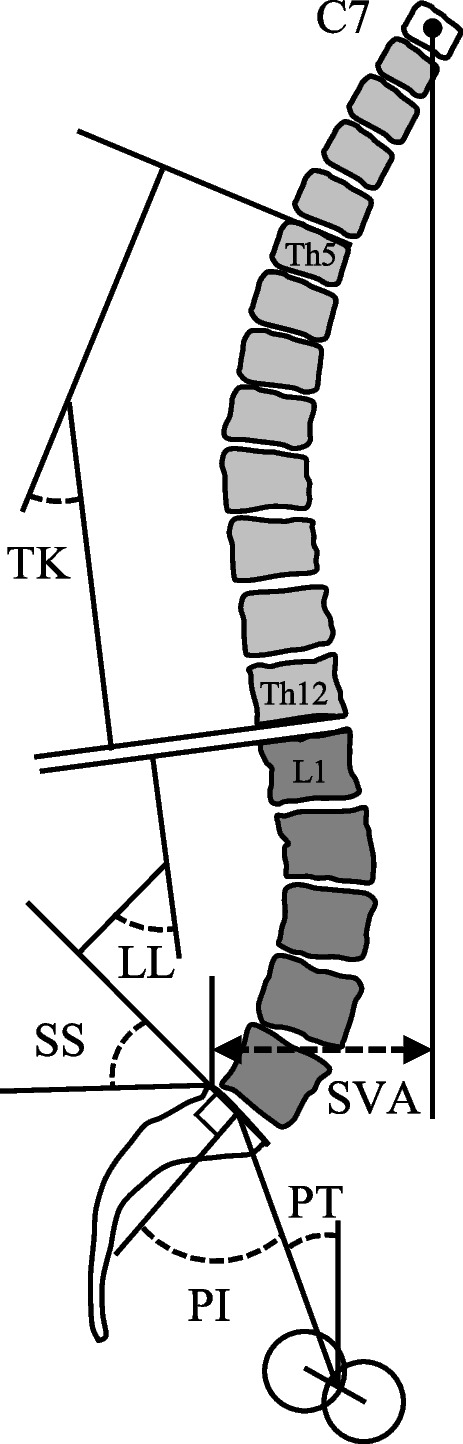
Sagittal spinal parameters assessed in this study. TK, thoracic kyphosis; LL, lumbar lordosis; SS, sacral slope; PI, pelvic incidence; PT, pelvic tilt; SVA, sagittal vertical axis

Preoperative radiographs included antero-posterior (AP) pelvic images and upright whole-spine images in the AP and lateral views. We reviewed the spine radiographs and evaluated the presence and level of VCF, and the following sagittal spinal parameters: C7 sagittal vertical axis (SVA), lumbar lordosis (LL), pelvic incidence (PI), pelvic tilt (PT), sacral slope (SS), and thoracic kyphosis (TK) (Fig. 2). Patients with low energy trauma or unrecognized VCFs were included. The definition of VCF on radiographs was based on the semiquantitative method proposed by Genant et al. [11]. We included grade 1 or higher, which were 20% or more reduction in vertebral height, and 10% or more reduction in vertebral area. All the vertebral bodies were reviewed using lateral whole-spine images. We classified all patients into with or without VCF (Fig. 1).

All THAs were performed by five senior hip surgeons using an anterolateral approach. The types of THAs were: hybrid, 356; uncemented, 82; and cemented, 15. The Harris hip score (HHS), Oxford hip score (OHS), University of California, Los Angeles (UCLA) activity score, and visual analog scale (VAS) score for LBP (0 = no pain; 10 = maximum pain) were recorded preoperatively and 1 year after THA.

### Statistical analyses

Two orthopedic surgeons (CC and YO) independently evaluated the preoperative whole-spine radiographs to diagnose VCFs. In cases of discrepancy between the two surgeons, another senior surgeon (TK) evaluated and determined the presence or absence of VCFs. The κ coefficient was calculated as the inter-observer reliability. The spinal parameters were measured by two surgeons. The intra-class correlation coefficients (ICCs) of inter-observer reliability were calculated for randomly selected 30 patients. Student’s t-test or the Mann–Whitney U test was performed to compare the variables between the two groups based on the results of the Shapiro–Wilk test. Fisher’s exact test was used to detect differences between the two groups on a nominal scale. All components of the HHS are shown as mean ± standard deviation, although they were ordinal scales and analyzed using the Mann–Whitney U test as it was difficult for readers to recognize the differences in data shown as median (interquartile range). Propensity scores were obtained from the logistic regression analysis using the following covariates: age, sex, body mass index (BMI), SVA, and PI-LL. We matched SVA and PI-LL to clarify the clinical relevance of VCFs in THA because these parameters have been reported to affect the clinical outcomes of THA [19]. A 1:1 propensity score in nearest-neighbor matching without replacement was performed to create matched pairs with the caliper set at 0.2 of a standard deviation of the logit of the propensity score. An absolute standardized difference (ASD) of < 0.20 was considered adequate. We further analyzed the demographics, sagittal spinal parameters, and pre-and postoperative changes in clinical assessments between patients with a single VCF and those with multiple VCFs. Statistical significance was set at *p* < 0.05. Statistical analyses were performed using the JMP Pro 15.0 software (SAS Institute, Cary, NC, USA).

## Results

The κ coefficient for the presence of VCF was 0.86, and the ICCs of the spinal parameters were greater than 0.85, indicating excellent consistency.

VCFs were identified in 51 (11.3%) patients. Patients with VCF were older, shorter, and had lower body weight than those without VCF (Table 1). Table 2 shows the relationship between the number of patients and VCFs. Single VCF was the most frequent, in 30 patients, and the most VCFs was four, in two patients. VCF occurred most frequently in the lumbo-thoracic transition areas of the Th12, L1, and L2 vertebrae (Table 3). Preoperative sagittal spinal parameters in patients with VCF showed lower LL and SS, larger PT and SVA, and greater PI-LL mismatch than those in patients without VCF, indicating sagittal spinal imbalance (Table 4). However, TK was higher in patients with VCF because of the kyphotic effects of VCFs on the thoracic vertebrae. Table 5 shows the clinical and VAS scores for LBP, which were significantly worse in patients with VCF, but not in those with postoperative OHS. The postoperative limp, support, distance walked, and stairs scores in the HHS were significantly poorer in patients with VCF. However, changes in the scores before and after THA were not significantly different between the groups.

**Table 1:** Demographics of all patients and propensity score-matched patients with and without vertebral compression fractures

	Patients with VCF	Patients without VCF	*p*-value	Matched patients with VCF	Matched patients without VCF	*p*-value	ASD
Number of patients	51	402		47	47		
Age (years)	71.9 ± 9.6	62.9 ± 12.2	< 0.01	71.2 ± 9.6	71.0 ± 7.4	0.90	0.02
Sex (M/F)^a^	7/44	72/330	0.56	7/40	8/39	1.00	0.06
Height (cm)	151.4 ± 6.6	156.1 ± 8.0	< 0.01	151.6 ± 6.79	153.6 ± 8.4	0.19	
Body weight (kg)	53.9 ± 11.2	58.2 ± 11.3	0.01	54.4 ± 11.6	57.4 ± 10.0	0.17	
Body mass index (kg/m^2^)	23.4 ± 4.2	23.8 ± 4.0	0.52	23.6 ± 4.36	24.3 ± 3.24	0.41	0.17
Diagnosis of the hip^a^			0.19			0.11	
OA	34	313		30	39		
ONFH	13	66		13	6		
Others (RDC, post-traumatic OA)	4	23		4	2		

Data are presented as mean ± standard deviation

*VCF* Vertebral compression fracture, *ASD* Absolute standardized difference, *OA* Osteoarthritis, *ONFH* Osteonecrosis of the femoral head, *RDC* Rapidly destructive coxarthrosis

^a^ Fisher’s exact test was performed

**Table 2:** Number of patients classified according to number of vertebral compression fractures

Number of vertebral compression fracture	Number of patients
1	30
2	14
3	5
4	2

**Table 3:** Spine level and number of vertebral compression fractures

Spine level	Number of vertebral compression fracture
Th5	1
Th6	3
Th7	1
Th8	2
Th9	3
Th10	3
Th11	8
Th12	16
L1	15
L2	11
L3	7
L4	5
L5	6

**Table 4:** Preoperative sagittal spinal parameters of all patients and matched patients with and without VCF

	Patients with VCF	Patients without VCF	*p*-value	Matched patients with VCF	Matched patients without VCF	*p*-value	ASD
Number of patients	51	402		47	47		
LL (°)	35.4 ± 21.7	45.5 ± 15.5	< 0.01	36.7 ± 20.2	35.9 ± 20.5	0.86	
PI (°)	51.9 ± 13.2	51.1 ± 11.6	0.62	51.2 ± 13.1	49.5 ± 11.2	0.49	
PT (°)	22.2 ± 12.3	15.0 ± 9.3	< 0.01	21.5 ± 12.0	18.5 ± 10.2	0.19	
SS (°)	29.7 ± 14.0	36.0 ± 10.5	< 0.01	29.7 ± 14.1	31.0 ± 12.9	0.65	
TK (°)	30.3 ± 14.3	26.0 ± 12.1	0.02	29.8 ± 14.5	27.2 ± 16.4	0.40	
SVA (mm)^a^	59.0 (31.2 − 107.7)	27.9 (6.6 − 59.7)	< 0.01	55.1 (29.8 − 96.6)	61.6 (31.2 − 92.8)	0.60	0.12
PI-LL (°)	16.6 ± 19.8	5.6 ± 14.8	< 0.01	14.6 ± 17.5	13.6 ± 18.7	0.79	0.07

Data are presented as mean ± standard deviation for normally distributed variables and median (interquartile range) for non-normally distributed variables

*VCF* Vertebral compression fracture, *ASD* Absolute standardized difference, *LL* Lumbar lordosis, *PI* Pelvic incidence, *PT* Pelvic tilt, *SS* Sacral slope, *TK* Thoracic kyphosis, *SVA* Sagittal vertical axis

^a^ Mann–Whitney U test was performed

**Table 5:** Results of clinical assessments and visual analog scale scores for low back pain

	Patients with VCF	Patients without VCF	*p*-value	Matched patients with VCF	Matched patients without VCF	*p*-value
Preoperative clinical assessments						
HHS	49.2 ± 13.0	54.8 ± 13.9	0.01	49.2 ± 13.5	56.4 ± 13.3	0.01
Pain^a^	15.6 ± 7.6	17.2 ± 8.6	0.09	15.8 ± 7.7	18.7 ± 8.0	0.02
Limp^a^	5.4 ± 3.1	5.7 ± 2.8	0.63	5.3 ± 3.1	5.4 ± 2.7	0.96
Support^a^	6.0 ± 3.3	7.3 ± 3.2	0.01	6.0 ± 3.3	7.2 ± 2.9	0.04
Distance walked^a^	5.2 ± 2.5	6.5 ± 2.5	< 0.01	5.2 ± 2.6	6.7 ± 2.5	< 0.01
Stairs^a^	1.7 ± 0.6	2.0 ± 0.6	< 0.01	1.7 ± 0.6	2.0 ± 0.4	0.047
Shoes and socks^a^	2.0 ± 1.1	2.3 ± 1.0	0.04	2.0 ± 1.1	2.3 ± 1.0	0.25
Sitting^a^	5.0 ± 0.3	5.0 ± 0.3	0.90	5.0 ± 0.3	5.0 ± 0.3	1.00
Public transport^a^	0.8 ± 0.4	0.9 ± 0.2	< 0.01	0.8 ± 0.4	0.9 ± 0.3	0.14
Deformity^a^	3.9 ± 0.6	3.8 ± 0.9	0.31	3.9 ± 0.6	3.9 ± 0.6	1.00
OHS	26.7 ± 11.8	31.3 ± 9.1	< 0.01	26.7 ± 11.3	29.9 ± 10.3	0.15
UCLA activity score^a^	3 (2–4)	4 (3–5)	< 0.01	3 (2–4)	3 (3–5)	0.055
VAS for LBP^a^	4 (0–7)	2.5 (0–5)	0.03	4 (0–7)	2 (0–4.5)	0.049
Postoperative clinical assessments						
HHS^a^	89.8 (82.8–96.9)	96.8 (90.7–99.8)	< 0.01	89.8 (81.8–97.0)	94.9 (87.7–99.7)	0.04
Pain^a^	42.8 ± 3.0	42.8 ± 3.3	0.95	42.8 ± 3.1	42.9 ± 2.5	0.85
Limp^a^	9.2 ± 2.0	10.0 ± 1.9	< 0.01	9.1 ± 2.0	9.7 ± 2.3	0.06
Support^a^	7.6 ± 2.9	9.6 ± 2.5	< 0.01	7.6 ± 2.9	9.2 ± 2.6	< 0.01
Distance walked^a^	8.4 ± 2.5	9.9 ± 2.0	< 0.01	8.4 ± 2.5	9.5 ± 2.3	0.02
Stairs^a^	2.5 ± 0.9	3.0 ± 1.0	< 0.01	2.5 ± 0.9	2.6 ± 1.1	0.38
Shoes and socks^a^	3.5 ± 1.0	3.6 ± 0.8	0.31	3.4 ± 1.0	3.6 ± 0.9	0.34
Sitting^a^	5.0 ± 0.0	5.0 ± 0.0	1.00	5.0 ± 0.0	5.0 ± 0.0	1.00
Public transport^a^	1.0 ± 0.1	1.0 ± 0.1	0.53	1.0 ± 0.1	1.0 ± 0.1	1.00
Deformity^a^	4.0 ± 0.0	4.0 ± 0.3	0.61	4.0 ± 0.0	3.9 ± 0.6	0.32
OHS^a^	47 (39–48)	47 (45–48)	0.08	47 (39–48)	47 (43–48)	0.52
UCLA activity score^a^	4 (3–5)	5 (5–6)	< 0.01	4 (3–5)	5 (4–6)	0.11
VAS for LBP^a^	1 (0–5)	0 (0–2)	0.01	1 (0–5)	0 (0–1)	0.02
Changes of clinical assessments						
ΔHHS	39.5 ± 11.9	38.8 ± 13.4	0.71	39.4 ± 12.3	35.7 ± 12.6	0.15
ΔOHS	16.5 ± 10.4	14.0 ± 8.8	0.07	16.5 ± 10.3	15.0 ± 9.2	0.46
ΔUCLA activity score	1 (1–2)	1 (0–2)	0.77	1 (0–2)	1 (0–2)	0.78
ΔVAS for LBP	1.6 ± 3.2	1.6 ± 3.0	0.86	1.6 ± 3.2	1.5 ± 3.0	0.90

Data are presented as mean ± standard deviation for normally distributed variables and as median (interquartile range) for non-normally distributed variables

All components of the HHS are shown as mean ± standard deviation, although they are ordinal scales and were analyzed using the Mann–Whitney U test

*VCF* Vertebral compression fracture, *HHS* Harris hip score, *OHS* Oxford hip score, *UCLA* University of California, Los Angeles, *VAS* Visual analog scale, *LBP* Low back pain

^a^ Mann–Whitney U test was performed

After propensity score matching for age, sex, BMI, SVA, and PI-LL, 47 patients with and without VCF were established. The matched variables were considered adequate according to the ASDs. No significant differences in spinal parameters were found between the groups after matching (Table 4). Compared to patients without VCF, those with VCF showed worse HHS, especially regarding support and distance walked, and worse VAS scores for LBP pre- and postoperatively. However, no significant differences were found in the pre- and postoperative OHS and UCLA activity scores. The changes in clinical scores were not significantly different between the two groups after matching (Table 5).

Table 6 shows the results of the comparison between patients with a single VCF and those with multiple VCFs, comprising 30 and 21 patients, respectively. The postoperative HHS and UCLA activity scores were significantly lower in patients with multiple VCFs.

**Table 6:** Demographics, spinal parameters, and clinical assessments of patients with single VCF and multiple VCFs

	Patients with single VCF	Patients with multiple VCFs	*p*-value
Number of patients	30	21	
Age (years)	71.7 ± 9.9	72.4 ± 9.4	0.69
Sex (M/F)^a^	4/26	2/19	1.00
Height (cm)	151.5 ± 7.1	150.8 ± 5.3	0.72
Body weight (kg)	55.5 ± 9.1	51.4 ± 13.4	0.20
Body mass index (kg/m^2^)	24.1 ± 3.2	22.5 ± 5.3	0.17
Diagnosis of the hip^a^			0.91
OA	21	13	
ONFH	7	6	
Others (RDC, post-traumatic OA)	2	2	
LL (°)	38.7 ± 22.8	33.2 ± 18.9	0.36
PI (°)	52.4 ± 12.5	52.6 ± 11.8	0.95
PT (°)	20.8 ± 14.3	24.2 ± 8.7	0.33
SS (°)	31.6 ± 15.1	28.4 ± 11.2	0.42
TK (°)	28.7 ± 14.2	33.2 ± 14.5	0.28
SVA (mm)^a^	60.8 (28.4–96.5)	59.2 (36.8–115.3)	0.25
PI-LL (°)	13.7 ± 21.5	19.4 ± 17.9	0.32
Preoperative clinical assessments			
HHS	49.0 ± 13.5	49.1 ± 12.6	0.99
OHS	25.8 ± 11.4	27.5 ± 12.3	0.60
UCLA activity score^a^	3 (2–4)	3 (2–5)	0.48
VAS for LBP^a^	3 (0–7)	5 (0.5–6.7)	0.43
Postoperative clinical assessments			
HHS^a^	90.4 (86.0–98.4)	85.9 (79.8–93.4)	0.04
OHS^a^	48 (41–48)	45 (37–48)	0.18
UCLA activity score^a^	4.5 (4–6)	4 (3–4.5)	0.04
VAS for LBP^a^	1 (0–4.3)	1 (0–5)	0.38
Changes of clinical assessments			
ΔHHS	41.6 ± 11.1	36.8 ± 12.9	0.17
ΔOHS	18.0 ± 9.1	14.9 ± 12.0	0.31
ΔUCLA activity score	1 (1–2)	1 (-0.5–2)	0.27
ΔVAS for LBP	1.5 ± 2.9	1.5 ± 3.6	0.94

Data are presented as mean ± standard deviation for normally distributed variables and median (interquartile range) for non-normally distributed variables

*VCF* Vertebral compression fracture, *OA* Osteoarthritis, *ONFH* Osteonecrosis of the femoral head, *RDC* Rapidly destructive coxarthrosis, *LL* Lumbar lordosis, *PI* Pelvic incidence, *PT* Pelvic tilt, *SS* Sacral slope, *TK* Thoracic kyphosis, *SVA* Sagittal vertical axis, *OA* Osteoarthritis, *HHS* Harris hip score, *OHS* Oxford hip score, *UCLA* University of California, Los Angeles, *VAS* Visual analog scale, *LBP* Low back pain

^a^ Mann–Whitney U test was performed

## Discussion

To the best of our knowledge, this is the first study to analyze the effects of VCF on the clinical outcomes of THA. Our study found that the approximately 11% of patients had VCF before THA, who were older, had sagittal spinal imbalance, and had worse clinical outcomes pre- and postoperatively. After propensity score matching, patients with VCF had worse HHS (*p* < 0.05), especially regarding support and distance walked, and worse VAS scores for LBP (*p* < 0.05), pre- and postoperatively. However, the changes in clinical scores were not significantly different between the groups, indicating that patients with VCFs showed similar improvement to patients without VCF. Furthermore, patients with multiple VCFs had lower clinical HHS and UCLA activity scores than those with a single VCF.

Patients with VCFs have sagittal spinal imbalance [5, 10, 12]. The VCFs and sagittal spinal parameters were closely related. Furthermore, Ochi et al. [19] reported that preoperative sagittal spinopelvic alignment affects hip function after THA. Therefore, we consider that a patient cohort with or without VCF after matching only for age, sex, and BMI, may mislead the results regarding whether VCFs or sagittal spinal imbalance affect the results. Our study featured propensity score matching for not only age, sex, and BMI but also spinal parameters to clarify the relationship between VCFs and clinical outcomes, regardless of sagittal spinal parameters. We found that VCFs independently affected the clinical outcomes of THA. Therefore, hip surgeons should assess not only spinal alignment but also the presence of VCFs, when spine radiographs are routinely examined.

Some studies showed that VCFs were associated with poor physical function [4, 14]. Similarly, vertebral deformities are associated with functional impairment [3, 22]. Our study revealed that the functional components of HHS, support and walking distance, were significantly lower in the VCF group, which is consistent with the results of previous studies. The other HHS components did not differ significantly between the two groups. Therefore, although patients with VCF may not show sufficient improvement in support and distance walked after THA, hip surgeons and patients with VCF can expect similar improvements in other components of the HHS as compared to patients without VCF.

The postoperative HHS and UCLA activity scores in patients with multiple VCFs were significantly poorer than those in patients with a single VCF, although no significant difference was noted between the two groups before THA. A previous study demonstrated that the number of VCFs correlated with the global sagittal alignment, which was further correlated with the Oswestry Disability Index and Short-Form-12 [12]. Another study found that community-dwelling women with multiple VCFs had poorer physical function, including slower walking speed, shorter chair stand time, and shorter functional reach than those with a single VCF [2]. The number of VCFs should also be evaluated to predict the clinical scores, activities of daily living, and HRQoL of patients.

VCFs are also closely related to sarcopenia [27, 30], with different criteria for sarcopenia [6, 8] such as poor physical performance. One study reported that patients with sarcopenia showed poor functional scores on THA pre- and postoperatively [29]. In our study, some patients with VCF also had sarcopenia, however, we did not investigate grip strength, which is a major criterion of sarcopenia. Therefore, clinical outcomes of THA may have been affected by sarcopenia. This is a limitation of this study; patients with sarcopenia or VCF overlap. In this study, we focused on VCFs, however, its definition remains complex. The identification of patients with sarcopenia among many THA candidates is difficult, whereas a spine radiograph is a relatively easy way to check VCFs to identify patients with poor functional outcomes.

This study has some limitations. First, this was a retrospective study performed at a single institution. Selection bias or other biases may have been present for THA in this study. Second, this study lacked data on medication, bone mineral density, bone metabolism markers, and diagnosis of sarcopenia, which may be covariates of the clinical outcomes of THA. The relationships between these factors should be investigated in future studies. Third, this study included a small number of patients with VCF and a 1-year follow-up period. A prospective, large-scale, long-term follow-up study is required to further clarify the association between VCF and THA.

## Conclusion

This study found that 11.3% of the patients had VCF before THA. HHS, especially regarding support and distance walked, and VAS for LBP were worse in patients with VCF preoperatively and 1-year postoperatively, after matching for age, sex, BMI, SVA, and PI-LL. However, the improvements in the clinical scores were similar between the two groups with or without VCF. Our findings suggest that hip surgeons should evaluate not only spinal alignment but also the presence of VCF before performing THA.

## Data Availability

The datasets analyzed in this study are available from the corresponding author upon reasonable request.

## Abbreviations

THA: Total hip arthroplasty
VCF: Vertebral compression fracture
HRQoL: Health-related quality of life
LBP: Low back pain
SVA: Sagittal vertical axis
LL: Lumbar lordosis
PI: Pelvic incidence
PT: Pelvic incidence
SS: Sacral slope
TK: Thoracic kyphosis
HHS: Harris hip score
OHS: Oxford hip score
UCLA: University of California, Los Angeles
VAS: Visual analog scale
BMI: Body mass index
ASD: Absolute standardized difference

## References

[1] Al-Sari UA, Tobias J, Clark E (2016). Health-related quality of life in older people with osteoporotic vertebral fractures: a systematic review and meta-analysis. Osteoporos Int.

[2] Arima K, Abe Y, Nishimura T, Okabe T, Tomita Y, Mizukami S (2017). Association of vertebral compression fractures with physical performance measures among community-dwelling Japanese women aged 40 years and older. BMC Musculoskelet Disord.

[3] Burger H, Van Daele PL, Grashuis K, Hofman A, Grobbee DE, Schütte HE (1997). Vertebral deformities and functional impairment in men and women. J Bone Miner Res.

[4] Cawthon PM, Blackwell TL, Marshall LM, Fink HA, Kado DM, Ensrud KE (2014). Physical performance and radiographic and clinical vertebral fractures in older men. J Bone Miner Res.

[5] Chau LTC, Hu Z, Ko KSY, Man GCW, Yeung KH, Law YY (2021). Global sagittal alignment of the spine, pelvis, lower limb after vertebral compression fracture and its effect on quality of life. BMC Musculoskelet Disord.

[6] Chen LK, Woo J, Assantachai P, Auyeung TW, Chou MY, Iijima K (2020). Asian working group for sarcopenia: 2019 consensus update on sarcopenia diagnosis and treatment. J Am Med Dir Assoc.

[7] Crowe JF, Mani VJ, Ranawat CS (1979). Total hip replacement in congenital dislocation and dysplasia of the hip. J Bone Joint Surg Am.

[8] Cruz-Jentoft AJ, Bahat G, Bauer J, Boirie Y, Bruyère O, Cederholm T (2019). Sarcopenia: revised European consensus on definition and diagnosis. Age Ageing.

[9] Ellenrieder M, Bader R, Bergschmidt P, Fröhlich S, Mittelmeier W (2015). Coexistent lumbar spine disorders have a crucial impact on the clinical outcome after total hip replacement. J Orthop Sci.

[10] Fechtenbaum J, Etcheto A, Kolta S, Feydy A, Roux C, Briot K (2016). Sagittal balance of the spine in patients with osteoporotic vertebral fractures. Osteoporos Int.

[11] Genant HK, Wu CY, van Kuijk C, Nevitt MC (1993). Vertebral fracture assessment using a semiquantitative technique. J Bone Miner Res.

[12] Hu Z, Man GCW, Kwok AKL, Law SW, Chu WWC, Cheung WH (2018). Global sagittal alignment in elderly patients with osteoporosis and its relationship with severity of vertebral fracture and quality of life. Arch Osteoporos.

[13] Jinbayashi H, Aoyagi K, Ross PD, Ito M, Shindo H, Takemoto T (2002). Prevalence of vertebral deformity and its associations with physical impairment among Japanese women: The Hizen-Oshima Study. Osteoporos Int.

[14] Johansson L, Sundh D, Nilsson M, Mellström D, Lorentzon M (2018). Vertebral fractures and their association with health-related quality of life, back pain and physical function in older women. Osteoporos Int.

[15] Kurtz S, Mowat F, Ong K, Chan N, Lau E, Halpern M (2005). Prevalence of primary and revision total hip and knee arthroplasty in the United States from 1990 through 2002. J Bone Joint Surg Am.

[16] Kurtz S, Ong K, Lau E, Mowat F, Halpern M (2007). Projections of primary and revision hip and knee arthroplasty in the United States from 2005 to 2030. J Bone Joint Surg Am.

[17] Maradit Kremers H, Larson DR, Crowson CS, Kremers WK, Washington RE, Steiner CA (2015). Prevalence of total hip and knee replacement in the United States. J Bone Joint Surg Am.

[18] Matsuoka H, Nanmo H, Nojiri S, Nagao M, Nishizaki Y (2021) Projected numbers of knee and hip arthroplasties up to the year 2030 in Japan. J Orthop Sci;10.1016/j.jos.2021.09.002 10.1016/j.jos.2021.09.002 34593285

[19] Ochi H, Homma Y, Baba T, Nojiri H, Matsumoto M, Kaneko K (2017). Sagittal spinopelvic alignment predicts hip function after total hip arthroplasty. Gait Posture.

[20] Ogino D, Kawaji H, Konttinen L, Lehto M, Rantanen P, Malmivaara A (2008). Total hip replacement in patients eighty years of age and older. J Bone Joint Surg Am.

[21] Okuzu Y, Goto K, Kuroda Y, Kawai T, Matsuda S (2022). Preoperative factors associated with low back pain improvement after total hip arthroplasty in a Japanese Population. J Arthroplasty.

[22] Pluijm SM, Tromp AM, Smit JH, Deeg DJ, Lips P (2000). Consequences of vertebral deformities in older men and women. J Bone Miner Res.

[23] Prather H, Van Dillen LR, Kymes SM, Armbrecht MA, Stwalley D, Clohisy JC (2012). Impact of coexistent lumbar spine disorders on clinical outcomes and physician charges associated with total hip arthroplasty. Spine J.

[24] Schousboe JT (2016). Epidemiology of Vertebral Fractures. J Clin Densitom.

[25] Singh JA (2011). Epidemiology of knee and hip arthroplasty: a systematic review. Open Orthop J.

[26] Sloan M, Premkumar A, Sheth NP (2018). Projected volume of primary total joint arthroplasty in the U.S., 2014 to 2030. J Bone Joint Surg Am.

[27] Takahashi K, Kubo A, Ishimura K, Fukui T, Tamura T (2018). Correlation among sarcopenia, malnutrition and activities of daily living in patients with vertebral compression fractures: a comparison based on admission and discharge parameters evaluating these conditions. J Phys Ther Sci.

[28] Toro G, Bothorel H, Saffarini M, Jacquot L, Chouteau J, Rollier JC (2019). Uncemented total hip arthroplasty in octogenarian and nonagenarian patients. Eur J Orthop Surg Traumatol.

[29] Ueoka K, Kabata T, Kajino Y, Inoue D, Ohmori T, Ueno T (2022). The prevalence and impact of sarcopenia in females undergoing total hip arthroplasty: a prospective study. Mod Rheumatol.

[30] Wang WF, Lin CW, Xie CN, Liu HT, Zhu MY, Huang KL (2019). The association between sarcopenia and osteoporotic vertebral compression refractures. Osteoporos Int.

